# Associating Cognition With Amyloid Status Using Partially Ordered Set Analysis

**DOI:** 10.3389/fneur.2019.00976

**Published:** 2019-09-13

**Authors:** Sarah J.A. Carr, Judith Jaeger, Shijia Bian, Ping He, Nancy Maserejian, Wenting Wang, Paul Maruff, Ahmed Enayetallah, Yanming Wang, Zhengyi Chen, Alan Lerner, Curtis Tatsuoka, Australian Imaging Biomarkers and Lifestyle Flagship Study of Ageing

**Affiliations:** ^1^Department of Neurology, Case Western Reserve University, Cleveland, OH, United States; ^2^Neuroimaging Department, Institute of Psychiatry, Psychology and Neuroscience, King's College London, London, United Kingdom; ^3^CognitionMetrics, LLC, Wilmington, DE, United States; ^4^Department of Psychiatry and Behavioral Sciences, Albert Einstein College of Medicine, Bronx, NY, United States; ^5^Biogen, Cambridge, MA, United States; ^6^Florey Institute of Neuroscience and Mental Health, University of Melbourne, Parkville, VIC, Australia; ^7^CogState Ltd., Melbourne, VIC, Australia; ^8^Department of Radiology, Case Western Reserve University, Cleveland, OH, United States; ^9^Neurological Institute, University Hospitals Cleveland Medical Center, Beachwood, OH, United States; ^10^Department of Population and Quantitative Health Sciences, Case Western Reserve University, Cleveland, OH, United States

**Keywords:** Alzheimer's disease, cognitive impairment, amyloid, partially ordered sets, ADNI/AIBL

## Abstract

**Background:** The presence of brain amyloid-beta positivity is associated with cognitive impairment and dementia, but whether there are specific aspects of cognition that are most linked to amyloid-beta is unclear. Analysis of neuropsychological test data presents challenges since a single test often requires drawing upon multiple cognitive functions to perform well. It can thus be imprecise to link performance on a given test to a specific cognitive function. Our objective was to provide insight into how cognitive functions are associated with brain amyloid-beta positivity among samples consisting of cognitively normal and mild cognitively impaired (MCI) subjects, by using partially ordered set models (POSETs).

**Methods:** We used POSET classification models of neuropsychological test data to classify samples to detailed cognitive profiles using ADNI2 and AIBL data. We considered 3 gradations of episodic memory, cognitive flexibility, verbal fluency, attention and perceptual motor speed, and performed group comparisons of cognitive functioning stratified by amyloid positivity (yes/no) and age (<70, 70–80, 81–90 years). We also employed random forest methods stratified by age to assess the effectiveness of cognitive testing in predicting amyloid positivity, in addition to demographic variables, and *APOE*4 allele count.

**Results:** In ADNI2, differences in episodic memory and attention by amyloid were found for <70, and 70–80 years groups. In AIBL, episodic memory differences were found in the 70–80 years age group. In both studies, no cognitive differences were found in the 81–90 years group. The random forest analysis indicates that variable importance in classification depends on age. Cognitive testing that targets an intermediate level of episodic memory and delayed recall, in addition to *APOE*4 allele count, are the most important variables in both studies.

**Conclusions:** In the ADNI2 and AIBL samples, the associations between specific cognitive abilities and brain amyloid-beta positivity depended on age, but in general episodic memory was most consistently predictive of brain amyloid-beta positivity. Random forest methods and OOB error rates establish the feasibility of predicting the presence of brain beta-amyloid using cognitive testing, *APOE*4 genotyping and demographic variables.

## Introduction

Novel therapies for early Alzheimer's Disease (AD) are in development which, if approved for clinical use, may increase the demand for confirmation of abnormally high levels of AD biomarkers such as brain beta-amyloid. Determination of brain beta-amyloid status using PET imaging of amyloid in the brain or CSF sampling is either expensive or invasive and therefore strategies that can increase confidence in decisions to order, or to not order such assessments will be very useful especially if they utilize information that can be obtained routinely. Amyloid identification is also of interest for AD clinical trial enrichment. Neuropsychological (NP) tests are often used to help providers diagnose AD, and therefore may also have an important role in predicting amyloid positivity. However, NP tests batteries used in AD are by design polyfactorial, in that multiple cognitive functions are measured to determine whether the nature and magnitude of any cognitive impairment observed is suggestive of dementia, and if so AD. As a result, it is difficult to link performance on a test to specific functions. Previous studies have investigated the cognitive profile associated with the presence of amyloid in AD dementia, MCI and cognitively unimpaired individuals using standard linear model approaches (1–7). Harrington et al. observed that among MCI cases, those who are amyloid positive vs. negative had greater deficit in verbal and visual memory (Hedge's G difference: 0.66 and 0.35, respectively) and attention/processing speed (Hedge's G = 0.31), but higher functioning in language (Hedge's G = −0.70) (1). However, results among cognitively unimpaired subjects have not been consistent across studies. For example, some studies did not observe statistically significant differences (1, 2), whereas other studies using larger sample sizes found associations between episodic memory and amyloid (3, 4, 7, 8). A meta-analysis of cognitively normal subjects with and without amyloid (9) found differences with small effect sizes for visuospatial function, processing speed, episodic memory, semantic memory, and executive function. Together, these findings indicate that the cognitive differences between amyloid positive and negatives in cognitively normal subjects can appear nuanced and difficult to detect.

Additionally, age of onset can also impact the cognitive profiles associated with AD (10). Patients with onset of AD at younger ages demonstrate praxis, language impairment and visuospatial problems, while older onset patients demonstrate a greater deficit in visual memory and temporal orientation (11, 12). In cognitively normal older adults, a decline of episodic memory has been well-studied and reviewed in Light (13), Tromp et al. (14). Additional deficits also may be apparent in attention, inhibition, cognitive flexibility (15) and processing speed (16).

One issue with these results is that tests are often grouped into subscales, and associated with certain functions. This approach requires replication within each scale, which is difficult given the time generally required to administer cognitive tests. The polyfactorial nature of tests leads to a reduction in internal consistency, which also hampers statistical power. Critically, it also complicates interpretation of scale scores.

The objective of this study is to characterize age-stratified cognitive profiles using NP test specificity among cognitively normal and MCI subjects, with the goal of identifying which cognitive abilities are most strongly related to amyloid biomarker measurements. In turn, this will aid in identifying promising targets for cognitive assessment in clinically practical amyloid detection tools. Focus is given to samples consisting of cognitively normal and MCI, since such subjects are likely to be targets for future AD treatments. We hypothesize that tailored and abbreviated sets of cognitive tests that are in line with these targets can help improve prediction of amyloid positivity. Given age and *APOE* genotype are important predictors of amyloid (2, 17–19), we explore stratification of cognitive testing by these variables.

To accomplish this, we applied partially ordered sets (POSET)-based statistical classification methods to NP test results from cognitively normal and MCI subjects. These data were obtained from the Alzheimer's Disease Neuroimaging Initiative (ADNI) database and the Australian Imaging, Biomarkers and Lifestyle Flagship Study of Aging (AIBL) database. We were able to select NP tests across the respective batteries that share the cognitive functions being assessed. This allowed for comparability of classification results even as the data sets were based on different test batteries, since the results are in relation to performance with the cognitive functions.

POSET statistical methods are classification models that “sift” through responses to polyfactorial NP measures and systematically identifies specific cognitive functions that are relatively impaired. Importantly, a theoretical statistical framework for this approach has been established (20, 21). POSETs have the ability to manage the aggregate response patterns and methodically identify specific functions that are the source of poor performance. For example, a Category Fluency test involves the person being asked to say all the words they can think of within a given semantic category, e.g., “vegetables.” The functions required for the test include verbal fluency, attention, and cognitive flexibility. If the individual performed badly on the test but performed well on another test, e.g., Boston Naming, which involves attention and verbal fluency, poor performance on Category Fluency could then be attributed to poor functioning in the domain of cognitive flexibility. Of course, variability in response behavior and the test score values themselves must be taken into account when assessing the strength of this evidence. This is formalized in a statistical Bayesian framework. Importantly, this systematic approach can be extended to more complex scenarios, when there are multiple functions involved within and across measures, as we will see here. Thus, POSETs have the ability to manage the aggregate response results and methodically classify cognitive profiles. POSET methods explicitly link functions and measures and provide a means for data-analytic validation of these links.

POSETs, implemented using custom written scripts in LUA programming language, have been successfully used in previous NP data studies to investigate cognitive profiles associated with progression from MCI to AD (22, 23). Baseline cognitive profiles of ADNI subjects were used to determine cognitive functions related to the risk of conversion from MCI to AD within 2 years. Deficits in specific levels of episodic memory level [recall of items after distraction as in the Auditory Verbal Learning Test List B (22)], perceptual motor speed, and cognitive flexibility were found to be potentially useful cognitive predictors of conversion. The presence of an *APOE*4 allele also had a strong association. Longitudinal change in specific functions was considered as well. POSETs have also been used in classifying schizophrenic cognitive profiles and functional recovery in schizophrenia (24, 25), and cognitive impairment patterns in low birthweight/early birth children at early grade school (26).

Overall, the objective of our POSET-based analyses was to provide insight into how cognition is associated with amyloid positivity, and to identify cognitive function targets for practical clinical decision tools to help predict amyloid presence. Focus was given to samples with primarily cognitively normal subjects, along with some MCI subjects. These samples are thus from potential screening populations. For the latter group, finding cognitive differences by amyloid status has been challenging in prior analyses (9). We then used random forest methods to assess the importance of individual cognitive tests in prediction.

## Methods

### ADNI and AIBL Studies

ADNI is a NIH funded study with the aim to advance understanding of AD through the use of repeated measurements over several years. The measurements include MRI, PET, clinical assessments, and NP tests. The data used in this study for the POSETs analysis were from the ADNI2 phase, and the information in the following sections describes the ADNI2 data only. For further information about the ADNI phases, see www.adni-info.org.

Similarly, AIBL was designed to investigate the factors that contribute to the development of AD. It focused largely on the recruitment of a cognitively normal and MCI population and is following them longitudinally. Data were collected by the AIBL study group, and the AIBL study methodology has been reported previously (27, 28). Further information about the AIBL study can be found on their website, see www.aibl.csiro.au.

The inclusion criteria for ADNI required participants to be aged 55–90 years old, have a minimum of 6 years of education, be fluent in either English or Spanish and have no other neurological conditions. AIBL inclusion criteria required participants to be aged ≥60 years of age and have no other neurological condition or a diagnosis of cancer, diabetes, or excessive regular consumption of alcohol. Both studies follow similar criteria for the diagnosis of dementia, based on the criteria of the National Institute of Neurological and Communicative Disorders and Stroke–Alzheimer's Disease and Related Disorders Association, and MCI based on the criteria proposed by Petersen et al. (29) [AIBL (27, 30), ADNI (31, 32)].

The ADNI group focused much more on recruiting MCI and early AD than in AIBL. To enhance comparability, we only consider normal, normal with no MCI diagnosis but with subjective memory concerns (SMC), and early MCI subjects in ADNI2, and only normal and MCI subjects in AIBL. Late MCI in ADNI2 have clear cognitive deficits, so would widen the variability in performance, and could impair comparability of the samples. Within the series of ADNI studies, we selected ADNI2 due to the consistent amyloid imaging, and careful characterization of early MCI subjects. The AIBL NP tests, although different, were designed to be comparable to the ADNI NP tests. We limit NP tests to those used in the same domains in a prior ADNI analysis (22), to allow for direct comparison.

### Analysis Samples

Participant data were selected from the ADNI2 databases if they had a status of cognitively normal controls, subjective memory concern but no MCI diagnosis (SMC) [see (32) for diagnostic criteria—henceforth considered with the normal controls] or early MCI; known amyloid status determined from PET imaging or CSF; *APOE*4 status recorded; and their NP battery test scores were available. AIBL subject data were selected for cognitively normal controls and MCI (which were not subdivided further, as in ADNI2). The demographics for each cognitive category are summarized in Tables 1A,B. The final ADNI2 sample had a total of 445 subjects consisting of 244 healthy adults (cognitively normal and subjective memory concern) and 201 MCI subjects. The group was 52% male, mean age was 71 6 years (*SD* = 6.5). *APOE*4 allele count was *APOE*4 = 0: 64.1%, *APOE*4 = 1: 31.3%, and *APOE*4 = 2: 4.3%. Amyloid status across the group was 40.0% positive. The final AIBL sample with amyloid imaging consisted of 210 subjects−175 healthy adults and 35 MCI subjects. The group was 50.4% male, mean age was 74.4 years (*SD* = 6.9), *APOE*4 allele count was *APOE*4 = 0: 67%, *APOE*4 = 1: 29.0%, and *APOE*4 = 2: 4.0%. Amyloid status across the group was 41.0% positive. Although the ADNI2 dataset contained more MCI subjects, the groups are comparable on age, gender, amyloid positivity, *APOE*4 allele count and cognitive status. The ADNI2 group did include a few subjects in the 55–60 years old range, and the AIBL subjects had a lower proportion of subjects with <13 years of education. Cognitive classifications were conducted for healthy (cognitively normal and SMC) and MCI subjects. Given that MCI subjects are more impaired and have higher likelihood of being amyloid positive, and since the mix of cognitively normal to MCI differs, we decided to conduct parallel analyses as opposed to pooling POSET classification results for combined analyses.

**Table 1:** ADNI2 and AIBL population demographics.

**ADNI2 (*N* = 445)**	**Cognitively normal**			**MCI**		
**(A)**						
Age range	<70 years	70–80 years	81–90 years	<70 years	70–80 years	81–90 years
Mean age (*SD*; years)	66.2 (2.5)	74.4 (3.1)	83.7 (2.0)	64.6 (3.7)	73.8 (2.9)	83.5 (2.1)
*N*	89	129	26	90	90	21
***APOE*****4 (%)**						
0	60.7	72.8	73.1	50.0	62.2	66.6
1	33.7	26.4	15.4	41.1	34.4	23.8
2	3.4	0.8	11.5	8.9	3.4	4.8
Unknown/not recorded	2.2	0.0	0.0	0.0	0.0	4.8
Amyloid positive (%)	23.6	34.9	46.2	41.1	50.0	61.9
Male (%)	40.4	49.6	65.4	52.2	62.2	47.6
Education ≥ 13 years (%)	86.5	93.0	84.6	93.3	81.1	61.9
**AIBL (*****N*** **=** **210)**	**Cognitively normal**			**MCI**		
**(B)**						
Age range	<70 years	70–80 years	81–90 years	<70 years	70–80 years	81–90 years
Mean age (*SD*; years)	66.4 (2.5)	74.8 (3.1)	84.7 (2.4)	65.0 (2.5)	75.1 (3.2)	83.4 (2.5)
*N*	52	97	26	6	18	11
***APOE*****4 (%)**						
0	67.3	72.2	73.1	66.6	38.9	54.5
1	32.7	23.7	26.9	16.7	44.4	36.4
2	0.0	4.1	0.0	16.7	16.7	9.1
Amyloid positive (%)	15.4	38.1	53.8	50.0	72.2	63.6
Male (%)	40.4	54.6	42.3	66.7	50.0	63.6
Education ≥ 13 years (%)	57.7	52.6	61.5	66.7	38.9	63.6

*(MCI, mild cognitive impairment, SD, standard deviation)*.

### Determination of Amyloid Status

In the ADNI study, the presence of amyloid was determined by the ADNI Biomarker and PET Cores from either CSF or PET data. PET data was acquired using the tracers Florbetapir (AV45) or fluorodeoxyglucose (FDG). Full details of the ADNI CSF and PET analysis protocols and their derivation of amyloid status can be found on the ADNI website and elsewhere (33, 34). For AIBL, the PET scans were analyzed by the AIBL Neuroimaging Research Stream. PET tracers used were Florbetapir (AV45), Flutemetamol, or the Pittsburgh compound B (PIB). Analysis details and the amyloid status determination details can be found in Rowe et al. (35) and on the AIBL website. In our analyses, we rely on PET analyses results to determine the presence of amyloid.

### Neuropsychological Data and Determination of Cognitive Functions

The ADNI2 and AIBL studies include a wide battery of NP tests. A selection of tests was chosen based on the types of cognitive functions they tested, as listed in Table 2. A variety of NP tests are needed to classify subjects to profiles of relative strengths and weaknesses across a range of functions for the POSET model. The final ADNI2 list includes Alzheimer's Disease Assessment Scale (ADAS)-Delayed Recall Subscale, ADAS-Word Recognition Subscale, Rey Auditory Verbal Learning Test (AVLT)-List 6, AVLT-List B, Boston Naming Test, Category Fluency, ADAS Number Cancellation, Trail Making Test A, and Trail Making Test B. The NP tests chosen from the AIBL study includes Boston Naming Test, California Verbal Learning Test—Delayed (CVLTD), CVLT—Recognition (CVLTR), CVLT—List B (CVLT List B), CVLT—Lists 1–5, category fluency, category switching, Stroop Word, Stroop Colors, CogState Detection, CogState Identification, CogState One back, and CogState One Card Learning. For both studies, given the range of ages, we derive age-normed test scores for analysis in the POSET models and random forests. This was conducted straightforwardly by obtaining z-scores that depend on age group within respective samples. Due to the wide age range, we consider stratifying analyses by 3 age groups: <70 years old (y), 70–80 years, and 81–90 years. Depending on the age of the subject, means and standard deviations from tests within the corresponding age group were used to derive z-scores. These age ranges were chosen based on expert clinical opinion (author AJL) and sample size balance across both studies. Standardized norming in the CVLT II Manual also is conducted in these age groups [see (36)].

**Table 2:** Selected neuropsychological tests from the ADNI2 and AIBL battery and their associated cognitive functions.

**NP test**	**Cognitive function**						
	**ATT**	**EM1**	**EM2**	**EM3**	**VF**	**CF**	**PS**
**ADNI2**							
ADAS delayed recall subscale	X	X	X	X			
ADAS word recognition subscale	X	X					
AVLT list 6	X	X	X	X			
AVLT list B	X	X	X				
Boston naming test	X				X		
Category fluency (animals and vegetables)	X				X	X	
ADAS number cancellation	X						
Trail making test A	X						X
Trail making test B	X					X	X
**AIBL**							
CVLT–delayed recall	X	X	X	X			
CVLT–word recognition	X	X					
CVLT–lists 1-5	X	X	X				
CVLT–list B	X	X	X				
Boston naming test	X				X		
Category fluency (animals and boy's names)	X				X	X	
Category switching	X				X	X	
Stroop–words	X						
Stroop–colors	X					X	
CogState–detection	X						X
CogState–identification	X						X
CogState–one back	X	X					
CogState–one card Learning	X	X	X				X

*ATT, attention; EM1, EM2, EM3, episodic memory level 1, 2, or 3; VF, verbal fluency; CF, cognitive flexibility; PS, perceptual motor speed*.

For our POSET analysis, experienced neuropsychologists developed a mapping of each NP test in ADNI2 (author JJ) and AIBL (authors JJ and PM) and the different cognitive functions being measured (Table 2). These mappings are validated data analytically (20), as we describe below. The cognitive functions included three levels of episodic memory, verbal fluency (VF), attention (ATT), cognitive flexibility (CF), and perceptual motor speed (PS). Three levels of episodic memory were distinguished to better represent the differences between immediate recall and delayed recall that are concealed in the aggregate scores of the NP tests (Table 3). Level 3 (EM3) is the highest level where subjects are able to recall given information at least 30 min later following a series of distractors. Level 2 (EM2) is the ability to recall information after a short duration (10 min), with distractors. For Level 1 (EM1), subjects are able to recall information immediately after receiving it but cannot recall it after a delay. These are hierarchically related; high-level performance on level 3 implies high-level performance at levels 1 and 2. Moreover, lower-level performance at level 1 implies lower-level performance at levels 2 and 3 as well. This ordered relationship between levels reduces the number of possible profiles, so, for instance, a subject cannot be high level at EM3 and low level at EM1.

**Table 3:** Description of episodic memory levels.

**Level**	**Description**
3	Recall longer term items with distractors, 30 min
2	Recall intermediate term items, such as AVLT list B, 10 min
1	Recall immediate term items, word recognition

### POSET Model Generation

Each NP test is associated with specific cognitive functions required to perform well on it. POSET models consist of classification states comprised of detailed profiles of cognitive functioning that reflect discrete performance levels across the range of associated functions (20, 21, 24, 25, 37). One state is considered higher than another state if its associated performance levels are at least as high for all cognitive functions as those of the lower one, and strictly greater for at least one of the functions. If neither state is higher than the other, in that each state is at a high level for a function that the other is not, then the two states are said to be incomparable. Allowing for incomparability enhances the flexibility to model response data. What is considered as high and low level is relative to within each study sample.

The respective POSET models are algorithmically generated based on the cognitive specifications of the tests, as in Table 2 (38). Equivalence classes of profiles are identified, where profiles are in a same equivalence class if they cannot be statistically distinguished by the test battery. Hence, the models are identifiable, and well-defined. The model is comprised of classification states that correspond to these equivalence classes. For profiles in a same equivalence class, the functions for which they differ in the profiles are considered as undetermined. For example, in Table 2, note that cognitive flexibility is always assessed in conjunction with other cognitive functions (in addition to attention). There are thus limitations in definitively distinguishing a subject's cognitive flexibility functioning levels. Specifically, when the other functions with which it is being tested are at low levels, performance on the respective tests is expected to be poor regardless of the functioning level of cognitive flexibility. Hence, its level cannot be determined in such cases. This phenomenon is termed as confounding in classification. In the model based on Table 2 on the ADNI2 specifications, the affected states were 7, 14, 21, and 28. Note that for these states, associated profiles indicate low levels for verbal fluency and perceptual motor speed. These are the two functions that are tested with cognitive flexibility, respectively, in Category Fluency and Trails Making Test B (20, 39). Hence, performance with cognitive flexibility is confounded in those situations, and its functioning level is undeterminable. In ADNI2 and AIBL, all tests were specified as involving Attention, so that bottom state profiles of 29 and 33, respectively, reflect that Attention is at a low level, and that due to confounding, the other function levels are undetermined. We assume that all functions are at low levels in these states. The AIBL state profiles do not have confounding beyond the bottom state. This is due to the larger variety of cognitive tests in the AIBL cognitive battery.

Partially ordered relationships between states allow for more flexibility and richness than linearly ordered models, and can represent the complex response patterns from NP tests. They also take advantage of replication in testing of function, as POSETs have essential statistical convergence properties such as accurately identifying a subject's cognitive profile with sufficient measurement. A main criterion for model fit involves assessing consistency in response behavior with respect to tests that are hierarchically related by the associated cognitive skill requirements. Additional information on POSET models is available elsewhere (20, 21, 40).

**Figure 1:**
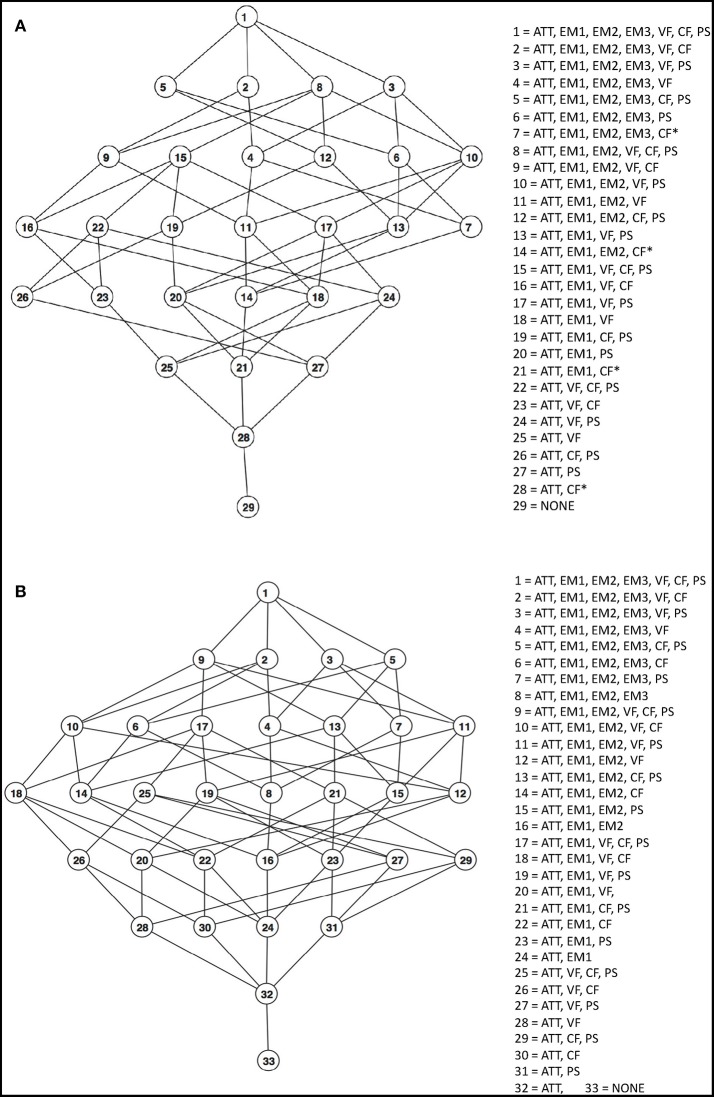
**(A)** ADNI2 POSET model for cognitive functioning. Attributes of each level are listed in more detail in Table 4A. **(B)** AIBL POSET model for cognitive functioning. Attributes of each level are listed in more detail in Table 4B. The cognitive functions listed represent high proficiency in those functions. ATT, attention; EM1, EM2, EM3, episodic memory level 1, 2, or 3; VF, verbal fluency; CF, cognitive flexibility; PS, perceptual motor speed.

The number of POSET states represents the number of possible cognitive profiles that can be determined from the selected NP tests, shown in Tables 4A,B for ADNI2 and AIBL, respectively. The associated cognitive functions were determined from expert opinion (JJ). The corresponding POSET models are shown graphically as Hasse diagrams in Figure 1, along with the profiles associated to each state. Listed are the functions at a high level. Profiles with undetermined CF functioning levels include CF^*^ in their respective list. Ordering of states is represented by level within a graph, with higher order states at higher levels in the graph. Direct connections between states indicate direct ordering, so that the upper connected state is at least as high a level for all functions as compared to the lower connected state, while also being at a higher level with at least one extra function. There were 29 POSET states in ADNI2 and 33 POSET states in AIBL in this analysis: the lowest state (number 29 and 33, respectively) possesses the lowest level of functioning across all cognitive functions, and the highest state (number 1 and 1) represents the highest level of functioning. Every state in between has at least one cognitive function that is at a low level. Thus, each state denotes a distinct cognitive profile. In Figures 1A,B, a line connecting two states indicates a direct ordering between a lower and higher state.

**Table 4:** ADNI2 POSET states and their relation to cognitive functions.

**POSET state**	**Cognitive function**						
	**ATT**	**EM1**	**EM2**	**EM3**	**VF**	**CF**	**PS**
**(A)**							
1	X	X	X	X	X	X	X
2	X	X	X	X	X	X	
3	X	X	X	X	X		X
4	X	X	X	X	X		
5	X	X	X	X		X	X
6	X	X	X	X			X
7	X	X	X	X		*	
8	X	X	X		X	X	X
9	X	X	X		X	X	
10	X	X	X		X		X
11	X	X	X		X		
12	X	X	X			X	X
13	X	X			X		X
14	X	X	X			*	
15	X	X			X	X	X
16	X	X			X	X	
17	X	X			X		X
18	X	X			X		
19	X	X				X	X
20	X	X					X
21	X	X				*	
22	X				X	X	X
23	X				X	X	
24	X				X		X
25	X				X		
26	X					X	X
27	X						X
28	X					*	
29							
**(B) AIBL POSET STATES AND THEIR RELATION TO COGNITIVE FUNCTIONS**							
1	X	X	X	X	X	X	X
2	X	X	X	X	X	X	
3	X	X	X	X	X		X
4	X	X	X	X	X		
5	X	X	X	X		X	X
6	X	X	X	X		X	
7	X	X	X	X			X
8	X	X	X	X			
9	X	X	X		X	X	X
10	X	X	X		X	X	
11	X	X	X		X		X
12	X	X	X		X		
13	X	X	X			X	X
14	X	X	X			X	
15	X	X	X				X
16	X	X	X				
17	X	X			X	X	X
18	X	X			X	X	
19	X	X			X		X
20	X	X			X		
21	X	X				X	X
22	X	X				X	
23	X	X					X
24	X	X					
25	X				X	X	X
26	X				X	X	
27	X				X		X
28	X				X		
29	X					X	X
30	X					X	
31	X						X
32	X						
33							

X denotes a high level of functioning has been assigned for a particular cognitive function in a particular POSET state.Within cognitive flexibility, *denotes an ambiguity exists and it is not possible to determine the contribution of this function. All else has been assigned a lower level function. ATT, attention; EM1, EM2, EM3, episodic memory level 1, 2, or 3; VF, verbal fluency; CF, cognitive flexibility; PS, perceptual motor speed.

Each POSET state was assigned a uniform prior probability value of 1/29 for ADNI2 and 1/33 for AIBL to indicate the non-informative prior belief about a participant's profile. Two response distributions were then estimated for each cognitive test, representing the statistical behavior of two groups: subjects in profiles with high level functioning for all the functions associated with a task, and those who are low level functioning in at least one of the associated functions. Based on a subject's set of responses, Bayes rule was used to update his or her posterior probabilities of state membership. See, for instance (20, 21), for details on how posterior probabilities are computed in Bayesian updating. A posterior probability value near one for a given state indicates that there is strong empirical evidence for the subject belonging to that state. In contrast, a value near 0 indicates that the subject likely does not belong to the state. Classification was conducted for cognitively normal and MCI subjects.

Response distribution estimation of the respective tests reflect tendencies for score values among subjects in the respective populations. Non-parametric approaches were adopted, as the shapes of the response distributions appeared complex (41). We categorized response values into four groups, demarcated by sample quartiles, so that multinomial response distributions are estimated in a Bayesian, Markov Chain Monte Carlo approach (24, 25). For the timed data from Trails A and B, Bayesian non-parametric density estimation using normal mixture models and Dirichlet process priors was employed (41). These estimated distributions are used in Bayes rule for computing posterior probabilities of state membership based on the relative likelihood of the observed responses and prior probabilities. Subjects will have relatively higher probabilities to perform well (e.g., upper quartile) on tests for which they have high level of functioning for all of the associated functions. Otherwise, it is expected that they perform less well with relatively higher probability.

Finally, for each subject, probabilities of being at a high level were derived for each cognitive function. These values were derived by summing posterior probability values of state membership with associated profiles that indicate high functioning with the specific function. Additional information is available in Tatsuoka (41).

### Statistical Comparison

Mann-Whitney *U* tests were used to compare by amyloid status the respective POSET-derived probabilities of high-level functioning across the range of cognitive functions. Comparisons were conducted within successively finer stratifications by age and number of *APOE*4 alleles. Age was stratified into three categories, <70 years, 70 up to 80 years and 81 up to 90 years. Bonferroni corrections were applied to the calculated *p*-values for the number of cognitive functions. Thus, the null hypothesis was rejected if the statistical significance surpassed a threshold of *p* < 0.05/7 = 0.007.

### Random Forests for Prediction of Amyloid Positivity With Cognitive Tests

We also developed example random forests based on respective cognitive tests in Table 2, in addition to age, gender, education (≥13 years or not), and *APOE*4 allele count. For ADNI2, we also include the MMSE total score (42). The objective is to assess the relative importance and utility for cognitive testing to predict amyloid status. This is done through analysis of classification error rates and variable importance measured with random forest methods. A key feature of random forests is the generation of an ensemble of classification trees based on bootstrapped samples of the data. Further, at each branch split in a tree, only a subset of randomly selected variables is considered. This helps reduce over-fitting. By classifying out-of-bag (OOB) data (about one-third of original sample) from each bootstrapped sample with the corresponding tree, an accurate estimate of tree-based classification error can be obtained without cross validation. Of great value is the measurement of variable importance in prediction by the mean decrease in accuracy (MDA) across an ensemble of trees by taking out each of the predictor variables individually from the tree fitting process, and assessing the resultant decrease in accuracy per tree. Below, *ntree* represents the number of trees that are fit per data set, and *mtry* is the number of variables randomly selected for each branch split (42). The R software package “randomForest” was used.

## Results

### Cognitive Differences by Amyloid and Age

POSET model fit in both models was good, as reflected by relatively large posterior probability values on one state, and response distribution estimates that reflect the specified order structure (20). Statistical tests stratified by age group revealed differences in cognitive profiles by amyloid status in both studies. In ADNI2, for the <70 years age group, the amyloid positives (A+) performed significantly worse than the amyloid negative (A−) group at EM1, EM2, EM3, and ATT. No significant differences were found in the AIBL <70 years group at the *p* < 0.007 threshold. For the 70 years up to 80 years age group in ADNI2, significantly worse performance for the A+ vs. A− group was observed for EM1, EM2 and ATT. This group in AIBL demonstrated significant differences in EM2. For study subjects aged 81–90 years, in both ADNI2 and AIBL, there were not significant differences between amyloid groups for any of the cognitive functions. The associated *p*-values for all the cognitive functions are shown in Table 5.

**Table 5:** Mann Whitney test *p*-values for cognitive difference by amyloid status, stratified by age.

**Age group**	**ATT**	**EM1**	**EM2**	**EM3**	**VF**	**CF**	**PS**	***n*+, *n*−**
**ADNI2**								
<70 years	0.001	0.001	0.004	0.003	0.073	0.121	0.376	57, 118
70–80 years	0.007	0.006	0.002	0.023	0.058	0.727	0.216	82, 123
81–90 years	0.894	0.771	0.489	0.796	0.594	0.816	0.197	33, 23
**AIBL**								
<70 years	0.083	0.211	0.097	0.038	0.381	0.348	0.274	13, 53
70–80 years	0.164	0.027	0.006	0.011	0.834	0.244	0.544	53, 67
81–90 years	0.037	0.016	0.032	0.028	0.843	0.368	0.615	30, 18

*n+, number of amyloid positive participants, n–, number of amyloid negative participants. ATT, attention; EM1, EM2, EM3, episodic memory level 1, 2, or 3; VF, verbal fluency; CF, cognitive flexibility; PS, perceptual motor speed*.

### *APOE*4 Allele Count, Age, and Amyloid

We next looked at how *APOE*4 and age predict amyloid status (see Table 6). In many instances, considering age and *APOE*4 allele count alone appears sufficient for prediction of amyloid status. For example, for *APOE*4 = 0 and age <70 years or 70–80 years in the ADNI2 sample, predicting amyloid status as negative would have led to accuracy levels of 81.8 and 71.8%, respectively. Also, for *APOE*4 = 1 and age 81–90 years, or *APOE*4 = 2 and age 70–80 years or 81–90 years, then respective prediction accuracies were 84.6, 75.0, and 100% for amyloid positive. For other age-*APOE*4 allele count groupings, accuracy is <70%. The AIBL sample revealed a similar picture of prediction accuracy with 89.5 and 77.6% negative in the <70 years and 70 up to 80 years groups for *APOE*4 = 0. For *APOE*4 = 1 and age 70 or older, accuracy remains high with 75.0% positive in the 70 up to 80 years group and 84.6% positive in the 81–90 years group. For *APOE*4 = 2, accuracy was 100% positive for all ranges, although sample sizes across the age groups are small.

**Table 6:** *APOE*4 allele count by amyloid status and age group.

	**Amyloid**	**<70 years**	**70–80 years**	**81–90 years**
**ADNI2**				
*APOE*4 = 0	Negative% (*n*)	81.8 (81)	71.8 (102)	53.8 (21)
	Positive	18.2 (18)	28.2 (40)	46.2 (18)
*APOE*4 = 1	Negative% (*n*)	50.8 (33)	33.9 (20)	15.4 (2)
	Positive	49.2 (32)	66.1 (39)	84.6 (11)
*APOE*4 = 2	Negative% (*n*)	36.4 (4)	25.0 (1)	0.0 (0)
	Positive	63.6 (7)	75.0 (3)	100.0 (4)
**AIBL**				
*APOE*4 = 0	Negative% (*n*)	89.5 (34)	77.6 (52)	60.0 (15)
	Positive	10.5 (4)	22.4 (15)	40.0 (10)
*APOE*4 = 1	Negative% (*n*)	64.7 (11)	25.0 (6)	15.4 (2)
	Positive	35.3 (6)	75.0 (18)	84.6 (11)
*APOE*4 = 2	Negative% (*n*)	0.0 (0)	0.0 (0)	0.0 (0)
	Positive	100.0 (1)	100.0 (5)	100.0 (3)

### Exploratory Analysis of Cognitive Differences by Amyloid, Age, and Cognitive Status

We next stratify by cognitive status (normal or MCI) as well as age group and amyloid status. This extra stratification results in smaller sample sizes and number of amyloid positive subjects per subgroup, hence we view these analyses as exploratory, and do not adjust for multiple comparisons. Mann-Whitney tests were adopted, to assess for differences across cognitive functions by amyloid status. Any differences found below indicate lower functioning for the amyloid positive group. Type I error level was set to 0.05.

For ADNI2 cognitively normal subjects, stratified by age group, the following cognitive functions had statistically significant differences between amyloid positive and negatives. For <70 years olds (*n* = 86, number of amyloid positives *n*+ = 21): VF (*p* = 0.044) and ATT (0.031), with EM3 trending toward significance (*p* = 0.102); for 70–80 years olds (*n* = 119, *n*+ = 40): ATT trended toward significance (*p* = 0.064); for 81–90 years olds (*n* = 32, *n*+ = 17): ATT trended toward significance (*p* = 0.105).

For ADNI2 MCI subjects, the following statistically significant differences in cognitive function were found: For <70 years olds (*n* = 90, *n*+ = 37): EM3 (*p* = 0.037), with EM2 trending toward significance (*p* = 0.068); for 70–80 years olds (*n* = 86, *n*+ = 42): episodic memory levels 1, 2, and 3 (respectively, *p* = 0.004; *p* = 0.012; *p* = 0.020); VF (*p* = 0.031), ATT (*p* = 0.009), CF (*p* = 0.038), with PS trending toward significance (*p* = 0.109); for 81–90 years olds (*n* = 24; *n*+ = 16): no significant differences.

For AIBL cognitively normal subjects, stratified by age group, there were no statistically significant differences across cognitive functions between amyloid positive and negatives. For <70 years olds, *n* = 51, *n*+ = 8; for 70–80 years olds, *n* = 83, *n*+ = 28; for 81–90 years olds, *n* = 27, *n*+ = 15. For AIBL MCI subjects, no differences were found for <70 years olds (*n* = 5, *n*+ = 3); for 70–80 years olds (*n* = 13, *n*+ = 10), trending toward significance was found for EM2 (*p* = 0.077); for 81–90 years olds (*n* = 14, *n*+ = 9), significant difference was found for EM1 (*p* = 0.042), and trends were seen for EM2 and EM3, and for ATT (*p* = 0.060 each).

### Random Forests for Predicting Amyloid Positivity and Assessing Variable Importance in Prediction

As we saw in the previous section, *APOE*4 allele count on its own is often quite predictive, depending on age, but not in all scenarios. Hence, this analysis will inform how cognitive tests can augment and improve prediction. Through assessment of variable importance, we can also ascertain prediction performance when *APOE*4 allele count is removed as a predictor through its mean decrease in accuracy (MDA) value. Given our interest in brief clinical assessment for screening, we consider individual cognitive tests, as opposed to classification at the cognitive function level, which would generally require replication in testing (see Table 7).

**Table 7:** Random forest results for ADNI2 and AIBL using all complete data records.

	**All data** **Importance of variables (*n* = 437, yes/no = 173/264)**	**MDA**				
**(A) ALL DATA INCLUDED FOR ADNI2 AND AIBL**						
ADNI2	*APOE*4	36.0				
	ADAS NC	16.9				
	Age	15.8				
	ADAS DR	11.2				
	AVLT list B	6.6				
	Gender	6.3				
	Trails B	4.3				
	Boston	4.2				
	AVLT list 6	1.8				
	ADAS WR	1.6				
	ED > 13 years	1.2				
	MMSE	0.1				
	Trails A	-2.0				
	Category Fl	-2.2				
	**(*****n*** **=** **234, yes/no** **=** **96/138)**					
AIBL	*APOE*4	33.5				
	Age	15.4				
	CVLT DR	14.7				
	CVLT list 1–5	13.7				
	CogState 1card	11.8				
	CogState 1back	10.8				
	CogState ID	5.3				
	Category switch	3.1				
	CVLT Recog	2.5				
	Category Fl	1.7				
	Stroop–Colors	1.5				
	Stroop–Words	1.4				
	Boston	1.3				
	CogState Det	-1.2				
	Ed > 13 y	-2.4				
	Gender	-2.6				
	**<70 years**		**70–80 years**		**81–90 years**	
	**Importance of variables (*****n*** **=** **176, yes/no** **=** **58/118)**	**MDA**	**Importance of variables (*****n*** **=** **205, yes/no** **=** **82/123)**	**MDA**	**Importance of variables (*****n*** **=** **56, yes/no** **=** **33/23)**	**MDA**
**(B) All DATA FOR ADNI2 AND AIBL, SUBGROUPED BY AGE**						
ADNI2	*APOE*4	20.9	*APOE*4	20.7	*APOE*4	17.2
	ADAS DR	13.3	ADAS DR	8.3	AVLT List 6	4.3
	Trails B	9.3	AVLT List B	7.9	Gender	3.4
	ADAS WR	9.0	Trails B	6.4	ADAS NC	2.8
	Gender	3.9	Boston	4.9	AVLT List B	2.7
	Boston	3.7	ADAS NC	3.8	MMSE	1.7
	AVLT list 6	3.5	Category Fl	3.5	Trails A	0.3
	ADAS NC	0.5	Ed > 13 y	2.0	Ed > 13 y	-0.4
	AVLT list B	0.4	Trails A	1.4	Trails B	-2.5
	Ed > 13 y	-0.2	Gender	1.3	Boston	-2.8
	MMSE	-0.3	ADAS WR	-0.2	Category Fl	-4.2
	Trails A	-3.2	MMSE	−0.8	ADAS WR	-4.3
	Category Fl	-5.2	AVLT list 6	-0.8	ADAS DR	-5.4
	**(*****n*** **=** **66, yes/no** **=** **13/53)**		**(*****n*** **=** **108, yes/no** **=** **44/64)**		**(*****n*** **=** **60, yes/no** **=** **39/21)**	
AIBL	CogState 1Card	11.8	*APOE*4	33.9	*APOE*4	8.8
	Stroop–Words	8.6	CVLT lists 1–5	16.4	CVLT DR	5.6
	Category Fl	5.3	CVLT DR	8.4	CVLT lists 1–5	5.2
	CVLT DR	5.0	CogState ID	5.9	Stroop–Words	4.7
	CogState 1Back	4.7	CogState 1Back	5.4	Stroop–Colors	2.4
	Stroop—Colors	2.6	Category Switch	5.2	CogState 1Back	1.9
	CVLT recog	1.6	Boston	3.9	CogState 1Card	0.9
	*APOE*4	0.8	Category Fl	2.3	CVLT Recog	0.7
	CogState Det	0.4	CogState 1Card	2.2	CogState ID	0.6
	CVLT lists 1–5	0.2	Gender	2.1	Category Fl	0.2
	Ed > 13 years	-0.1	Stroop–Colors	0.6	Category switch	0.1
	Gender	-0.6	CVLT Recog	-0.9	Gender	-0.1
	Category switch	-0.7	CogState Det	-0.9	CogState Det	-2.5
	CogState ID	-1.0	Ed > 13 y	-1.2	Ed > 13 y	-2.6
	Boston	-1.3	Stroop–Words	-1.8	Boston	-3.5

*Yes/no, amyloid positive/negative; ADAS DR, ADAS Delayed Recall; ADAS WR, ADAS Word Recognition; ADAS NC, ADAS Number Cancellation; Trails A; Trails Making test A; Trails B, Trails Making test B; Boston, Boston Naming Test; MMSE; Mini Mental State Exam total score; Category Fl, Category Fluency Test; Ed > 13 years; Education > 13 years, CVLT DR, CVLT Delayed Recall; CVLT Recog, CVLT Recognition; CogState ID, CogState Identification; CogState 1Card, CogState One Card Learning; CogState 1Back, CogState One Back and CogState Det, CogState Detection. MDA, Mean Decrease in Accuracy*.

Four variables were randomly selected per branch split (mtry = 4, ntree = 1,000). The random forest OOB error rates using all data in ADNI2 and AIBL are 30.88 and 27.14%. The OOB error rates in the age subgroups <70, 70–80, 81–90 years were 30.46, 32.35, and 30.36% in ADNI2, and 17.24, 24.76, and 4.43% in AIBL. For AIBL, the OOB error rate worsens for the older age groups. The relative ranking of variable importance and associated mean decrease in accuracy values are interesting. Note that in ADNI2, we see that *APOE*4 is the most important variable in prediction. In the random forest with all data, not including *APOE*4 allele count as a variable results in a 36% mean decrease in accuracy. This indicates that cognitive testing alone may not be as effective in overall prediction without *APOE*4 allele status. Note that ADAS Number Cancellation, a measure of attention, has the second highest importance, followed by age and ADAS Delayed Recall. The cognitive tests are in alignment with our POSET-based findings that differences in ATT and EM3 are statistically significant when age is <80 years.

For AIBL, *APOE*4 allele count is by far the most important variable overall for the random forest fit with all the subjects together. Still, note that a number of episodic memory tasks also have relatively high importance (e.g., <10% mean decrease in accuracy), as well as the age variable. This includes CVLT Delayed Recall and List 1–5 tasks, and CogState One Card and One Back tasks. *APOE*4 allele count is important for the 70–80 and 81–90 years age groups as well, but interestingly, it is not relatively important in the <70 years age group. In that age group, CogState One Card is the most important variable, and the only one with <10% MDA. In this subgroup, note that the number of positive amyloid subjects is relatively small, which may be a factor in the low OOB error rate. For the 70–80 years random forest, note that CVLT Lists 1–5 has MDA of 16.4%. This test is associated with EM2.

## Discussion

We applied POSET models to NP test scores from ADNI2 and AIBL to examine performance in a range of cognitive functions and characterize cross-sectional cognitive function deficit patterns that were associated with the presence of amyloid. These results showed that specific cognitive abilities differed by amyloid status and depended on age. In general, episodic memory, particularly intermediate recall with distraction (EM2), as well as delayed and immediate recall abilities (EM3 and EM1) and attention (ATT) most consistently emerged as being associated with amyloid positivity. These differences depend on age group. In ADNI2, for subjects <70 years old, cognitive differences by amyloid group are clear for EM1–EM3 and ATT even at the strict Bonferroni-corrected threshold of *p* < 0.007. These differences persist for the 70–80 years group, although EM3 differences are significant only at the *p* < 0.05 threshold. In AIBL, there are less clear differences, with only EM2 being significant at the stricter threshold for the 70–80 years group. However, at the *p* < 0.05 threshold, differences arise for EM3 at all age groups, and EM1 for the 70–80 years and 81–90 years groups. At that significance level, ATT is significantly different for the 81–90 years group.

Hence, the cognitive functions with differences between amyloid groups are similar across studies. The differences do appear to arise earlier in the ADNI2 cohort. The differences are also more decisive, in terms of smaller *p*-values, in ADNI2. This could be due to larger sample size, and the higher level of inclusion of MCI, so that differences with cognitively normal subjects is more pronounced. Interestingly, for ADNI2, there are no differences at either significance threshold in the 81–90 years group. This could be in part due to increases in other causes of cognitive impairment with aging leading to a reduction in cognitive variability among the oldest participants.

In section APOE4 Allele Count, Age, and Amyloid, it is interesting to see how well-amyloid positivity is predicted from *APOE*4 allele count, in conjunction with age group, in both studies. For instance, when *APOE*4 = 0 and age is <70 years then amyloid status is likely negative. In contrast, when *APOE*4 = 1 and age is >70 years then amyloid status is likely positive. When *APOE*4 = 2, amyloid status appears to be decisively positive. For other *APOE*4/age combinations, amyloid status is less clear. These results are in line with a prior study that found that an estimated 91% of people with two *APOE*4 alleles develop AD, at the average age of onset of 68 years, compared with just 47% for a single *APOE*4 allele, with average onset of 76 years (43). Carriers of the *APOE*4 gene also demonstrated a higher degree of cognitive decline (44).

The random forest results indicate that reasonable performance in prediction of amyloid positivity is possible with cognitive tests, *APOE*4 and demographic variables. Importantly, random forest variable importance results by age group give a sense of how importance changes with age. In ADNI2, *APOE*4 is still most important, and delayed recall measures are second most important. However, in the age group 81–90 years, the MDA for AVTOT6, the second most important variable, is only 4.3%, which indicates that cognitive testing may not be helpful for prediction in this age subgroup. For the 81–90 years age group random forest for AIBL, OOB error rate is 40.43%, indicating that even with *APOE*4 genotype, cognitive tests, and demographic variables, prediction of amyloid may be difficult in the age group. In both studies, it appears that for the 81–90 years age group, cognitive tests do not have high variable importance for predicting amyloid positivity.

Overall, the POSET and random forest analyses have strong correspondence. By age group, the cognitive functions identified as differing by amyloid status are also associated with the cognitive tests found to have relatively high variable importance. The POSET analysis provides scientific support for the selection of cognitive tests for prediction, and correspond to known cognitive sequalae in AD progression. A practical ramification of the POSET analyses is the focus at the cognitive function level, as opposed to the individual test level, as done in random forests. Although different cognitive test batteries were adopted, the cognitive test importance values are unified across studies by sharing common specifications for functions that significantly differ by amyloid status. This holds promise for flexibility in future screening in terms of utility of a range of cognitive tests that can be effective in prediction. Considerations of cost and burden of NP tests are important before implementing classification tree algorithms for practical clinical use. Time is often a limiting factor in clinics and conducting cognitive tests can be time consuming. For example, the ADAS–Delayed Recall Subscale takes roughly 30 min to complete for assessment of the EM3 function. Note that CogState memory tests, which are computerized, were also found to be important. It may thus be possible to streamline administration of tests to minimize impact on clinical flow and test burden.

We also conducted exploratory subgroup analyses by age and cognitive status in section Exploratory Analysis of Cognitive Differences by Amyloid, Age, and Cognitive Status. In many cases, sample sizes and the number of amyloid positives within the subgroup were often small. Still, there are interesting findings for cognitively normal subjects in the ADNI2 study for <70 years olds, with verbal fluency and attention being significantly different. For the MCI subjects in both studies, it appears episodic memory levels and other functions may be impacted, depending on age group. In prior studies, the differences in cognitive function in cognitively normal and MCI cohorts by amyloid presence have varied: some studies have reported no differences in the cognitive performance between healthy A+ and healthy A− groups (1, 2), while other studies have reported differences in episodic memory (7, 8, 45). It is difficult to compare the type (level) of episodic memory deficit between these studies and ours as they generally do not break the cognitive functioning down into the levels used here. Episodic memory has generally been reported as encompassing EM1, EM2, and EM3. Using data from AIBL, Lim et al. (2) observed that the cognitively normal A+ group had a subtle lower performance across all NP tests examined, compared to their cognitively normal A− group. In Tatsuoka et al. (22), it was found that POSET values of cognitive functioning were fairly effective for predicting conversion from MCI to AD within 24 months. EM2 was found to be the most promising of all the cognitive functions, in conjunction with *APOE*4 status. EM3 was less effective than EM2, perhaps owing to aging confounding, as some non-converters had poor EM3 functioning as well. These findings are not inconsistent with what we have found in the current analysis.

The AIBL MCI cohort at age 70–80 years old has a higher rate of amyloid positivity (72.2%) compared to the same group in ADNI (50.0%). Although this AIBL group also has a lower percentage with education duration <13 years (38.9 v 81.1%), the random forest analysis results show only a weak association with education for inferring the presence of amyloid. The higher rate of amyloid positivity may be explained by the higher incidence of *APOE*4 alleles in this group−44.4 and 16.7% for 1 and 2 alleles, respectively. The contemporary group in ADNI has the following rates−34.4 and 3.3%, respectively. This discrepancy may also be due to our restriction to early MCI in the ADNI2 group, to reduce the proportion of MCI in the sample, and to select less affected subjects. The MCI subjects in AIBL were not characterized as early or late, and hence are likely more heterogeneous in terms of their MCI stage.

Limitations of this analysis include the sample size reductions that resulted from the age group stratification. The posterior probability values being analyzed were highly non-normally distributed, so non-parametric methods along with subgroup stratification (age, *APOE*4 count, cognitive status) was adopted rather than linear models with covariate adjustment. Also, the ADNI2 and AIBL populations are somewhat clinically and demographically narrow, which makes it difficult to draw generalizable conclusions that can be applied to other populations. The ADNI2 sample has a high proportion of participants with MCI, many years of education, and is composed of a mostly Caucasian sample. The AIBL sample has less MCI but also has relatively lower levels of education, and also is mostly Caucasian. Note that education has been found to have a relatively weak association with memory decline, and so it appears to be an important marker in both studies (46). Also in AIBL, non-amnestic MCI were included in the dataset; this is not normally associated with progression to AD and may represent different underlying pathology.

We acknowledge that both amyloid and tau could play important roles in AD pathology. The notion that amyloid pathology defines AD has remained largely intact through each successive update to the diagnostic criteria. The amyloid hypothesis predicts that the neurofibrillary tangles and other disease-associated pathologies, including synapse degeneration, hippocampal atrophy and neuroinflammation, are downstream of amyloid pathology and less disease specific. Therefore, if an individual presents with positive amyloid, the current view is that it is consistent with the AD diagnosis criteria, and tau and/or neurodegeneration markers positivity is not necessary. This is a “consensus” view broadly shared by both the NIA-AA diagnostic guidelines (2011–2018) and the International Work Group (IWG) criteria (2007–2014). In our study, we followed the diagnosis criteria, and did not apply the tau positivity status in defining our study population. We acknowledge that the evidence of tau accumulation may help to address the heterogeneity of the study population in terms of AD pathologies. It should be noted that recent guidelines published by the NIA-AA include amyloid, tau and neurodegeneration in a recommended research framework of diagnosing AD (47). However, for these analyses, we chose to keep to the recommended clinical criteria.

Finally, we note that the random forest analysis was for illustrative purposes only and not for clinical use.

## Conclusion

These findings give insight into how specific aspects of cognitive functioning were associated with amyloid positivity, depending on age, in different samples comprised of cognitively normal, and MCI subjects. These samples represent potential screening populations. Through POSET models of ADNI2 and AIBL NP test data, cognitive functions were identified as targets for testing to help predict brain amyloid-beta positivity. Note that this approach is a more general approach than selection of specific tests, as it suggests the possibility that different cognitive tests can be useful in prediction, as long as they tap into the same cognitive function targets. Indeed, this is what was observed across the ADNI2 and AIBL studies, which adopted different test batteries. The analyses presented here showed that cognitive testing of intermediate and delayed recall (EM2 and EM3) may be particularly useful, as well as attention (ATT). They also indicate that for older subjects (81–90 years), prediction can be more difficult, even with cognitive tests. This finding is reflected in both ADNI2 and AIBL data sets. These results inform a potential role of cognitive testing in the development of clinical screening tools that inform prediction of amyloid positivity without the use of invasive and expensive approaches such as amyloid PET. The random forest analyses across the two studies suggest that abbreviated cognitive testing that focuses on these respective targets can still lead to moderately high prediction accuracy. Future work will focus on developing efficient and practical classifiers that can be used in clinical settings.

## Data Availability

The ADNI data that support the findings of this study are available from the ADNI repository, http://www.adni-info.org. The AIBL data that support the findings of this study are available from the AIBL repository, see www.aibl.csiro.au for further details.

## Ethics Statement

The ADNI study was conducted according to Good Clinical Practice guidelines, the Declaration of Helsinki, US 21CFR Part 50—Protection of Human Subjects, and Part 56—Institutional Review Boards, and pursuant to state and federal HIPAA regulations. Each participating site obtained ethical approval from their Institutional Review Board before commencing subject enrolment. Written informed consent was obtained from all subjects and/or authorized representatives and study partners before protocol-specific procedures were carried out. The AIBL study was conducted according to the Declaration of Helsinki and ethical approval was obtained for each participating site from their institutional ethics committees prior to commencement of enrolment. All participants gave written informed consent before engaging in study protocols. Ethical approval for this study for the analysis of ADNI and AIBL anonymized data was not required.

## Author Contributions

SC: analysis and interpretation of data and drafting of manuscript. JJ, SB, PH, NM, WW, and PM: interpretation of data and drafting of manuscript. AE: study design and interpretation of data. YW and AL: interpretation of data. ZC: analysis and interpretation of data. CT: study design, analysis and interpretation of data, and drafting of manuscript. All authors read and approved the final manuscript.

### Conflict of Interest Statement

SC, JJ, AL, YW, and CT were funded by Biogen to conduct this study. JJ was employed by company CognitionMetrics LLC. PM was employed by company CogState Ltd. SB, PH, NM, WW, and AE were employed by company Biogen. The remaining author declares that the research was conducted in the absence of any commercial or financial relationships that could be construed as a potential conflict of interest.
